# An Adapted Deep Convolutional Neural Network for Automatic Measurement of Pancreatic Fat and Pancreatic Volume in Clinical Multi-Protocol Magnetic Resonance Images: A Retrospective Study with Multi-Ethnic External Validation

**DOI:** 10.3390/biomedicines10112991

**Published:** 2022-11-21

**Authors:** John Zhiyong Yang, Jichao Zhao, Reza Nemati, Xavier Yin, Kevin Haokun He, Lindsay Plank, Rinki Murphy, Jun Lu

**Affiliations:** 1 School of Science, Faculty of Health and Environmental Sciences, Auckland University of Technology, Auckland 1010, New Zealand; 2 Auckland Bioengineering Institute, University of Auckland, Auckland 1142, New Zealand; 3 Canterbury Health Laboratories, Canterbury District Health Board, Christchurch 8022, New Zealand; 4 Department of Electrical Engineering and Computer Sciences, The University of California, Berkeley, CA 94720, USA; 5 School of Engineering and Applied Science, University of Pennsylvania, Philadelphia, PA 19104, USA; 6 School of Medicine, Faculty of Medical and Health Sciences, University of Auckland, Auckland 1142, New Zealand; 7 Auckland Diabetes Centre, Auckland District Health Board, Auckland 1142, New Zealand; 8 Whitiora Diabetes Department, Counties Manukau District Health Board, Auckland 1640, New Zealand; 9 Maurice Wilkins Centre for Molecular Biodiscovery, Auckland 1142, New Zealand; 10 School of Public Health and Interdisciplinary Studies, Faculty of Health and Environmental Sciences, Auckland University of Technology, Auckland 0627, New Zealand; 11 Institute of Biomedical Technology, Auckland University of Technology, Auckland 1010, New Zealand; 12 College of Food Engineering and Nutrition Sciences, Shanxi Normal University, Xi’an 710119, China; 13 College of Food Science and Technology, Nanchang University, Nanchang 330031, China

**Keywords:** pancreas segmentation, diabetes prognosis, organ fat measurement, machine learning, deep convolutional neural network (DCNN)

## Abstract

Pancreatic volume and fat fraction are critical prognoses for metabolic diseases like type 2 diabetes (T2D). Magnetic Resonance Imaging (MRI) is a required non-invasive quantification method for the pancreatic fat fraction. The dramatic development of deep learning has enabled the automatic measurement of MR images. Therefore, based on MRI, we intend to develop a deep convolutional neural network (DCNN) that can accurately segment and measure pancreatic volume and fat fraction. This retrospective study involved abdominal MR images from 148 diabetic patients and 246 healthy normoglycemic participants. We randomly separated them into training and testing sets according to the proportion of 80:20. There were 2364 recognizable pancreas images labeled and pre-treated by an upgraded superpixel algorithm for a discernible pancreatic boundary. We then applied them to the novel DCNN model, mimicking the most accurate and latest manual pancreatic segmentation process. Fat phantom and erosion algorithms were employed to increase the accuracy. The results were evaluated by dice similarity coefficient (DSC). External validation datasets included 240 MR images from 10 additional patients. We assessed the pancreas and pancreatic fat volume using the DCNN and compared them with those of specialists. This DCNN employed the cutting-edge idea of manual pancreas segmentation and achieved the highest DSC (91.2%) compared with any reported models. It is the first framework to measure intra-pancreatic fat volume and fat deposition. Performance validation reflected by regression R^2^ value between manual operation and trained DCNN segmentation on the pancreas and pancreatic fat volume were 0.9764 and 0.9675, respectively. The performance of the novel DCNN enables accurate pancreas segmentation, pancreatic fat volume, fraction measurement, and calculation. It achieves the same segmentation level of experts. With further training, it may well surpass any expert and provide accurate measurements, which may have significant clinical relevance.

## 1. Introduction

Diabetes is a glucose metabolism disorder, an increasingly prevalent chronic disease [1], with high morbidity and mortality that can be reduced by early diagnosis and treatment [2]. The pancreas plays a critical physiological role in maintaining glucose homeostasis. Recently, mendelian randomization studies have documented a causal role for pancreatic fat (and volume) in type 2 diabetes [3], after several reports describing an association between reduced pancreas volume and type 2 diabetes [4,5,6,7], or an increase in ectopic fat deposition in the pancreas in individuals with T2DM [8,9,10,11,12,13,14]. Thus, precise characterization of the pancreas can potentially improve diabetes risk stratification and treatment selection. Pancreatic fat can be evaluated by ultrasonography (US), computed tomography (CT), magnetic resonance imaging (MRI), and magnetic resonance spectroscopy (MRS). US and CT are now the first-tier measuring method. However, the US cannot quantify the entire length of the pancreas due to its relatively poor sonographic window; especially for those used in obese patients, CT has lower response sensitivity to the fat tissue gradient in the pancreas [15,16]. In contrast, MRI offers higher-sensitivity mechanisms for identifying fat from lean tissues based on T_1_ relaxation chemical-shift properties.

Over the past 20 years, the development of reconstruction algorithms, such as proton density fat fraction mapping (PDFF), has dramatically improved MRI techniques and enabled advanced fat fraction quantification functionality [17]. PDFF provides a reliable fat fraction result by exploiting the difference in resonance frequencies of protons in water and fat [18], which is currently regarded as the most valuable and meaningful MR-based biomarker of tissue fat fraction [19]. However, quantifying fat deposition inside relatively small organs such as the pancreas by MRI-PDFF is still challenging as it is based on delineating tissues entirely within the pancreas. The relative softness of the pancreas makes it easy to be squeezed by its surrounding organs, which reduces the demarcation of the boundaries of the pancreas, which then collapse with other non-pancreatic soft tissues, such as the small intestine, blood vessels, and visceral adipose tissues of the abdomen. Consequently, measurements of pancreatic volume and fat deposition by MRI in humans are highly variable [20,21,22,23]. Manual fat segmentation on MRI images by experienced evaluators is regarded as a gold standard [24]. To reduce inter-observer and intra-observer variability, one manual pancreatic fat measuring method quantifies the regions of interest (ROI) within the entire pancreas and excludes pixels with fat percentage values of <1% and >20%, which represent histological verified blood vessels, ducts, or visceral fat (termed MR-opsy) [24]. However, manual segmentation is still time-consuming.

The rapid development of Deep Convolutional Neural Networks (DCNNs), such as the full convolutional network (FCN) and conditional random fields (CRF) [25,26,27,28,29], has advanced much of the medical image analysis by enabling computers to learn organ segmentation tasks from large MRI datasets including heart, liver, kidney, and spleen [30,31]. However, automatic pancreas segmentation has rarely been reported to date due to the high inter-observer variation of this retroperitoneal organ. Furthermore, additional information shown from 3D MRI scans, compared to 2D images, makes it more complicated to establish 3D models by using the classical Atlas model [32] for context learning, due to the limitation of current GPU memory. While there are flaws in each method, updated 2D and 3D networks have been developed, and strategies such as coarse-to-fine framework and recurrent and ensemble learning have been applied to improve the accuracy of segmentation results [33,34,35,36]. 

**Figure 1:**
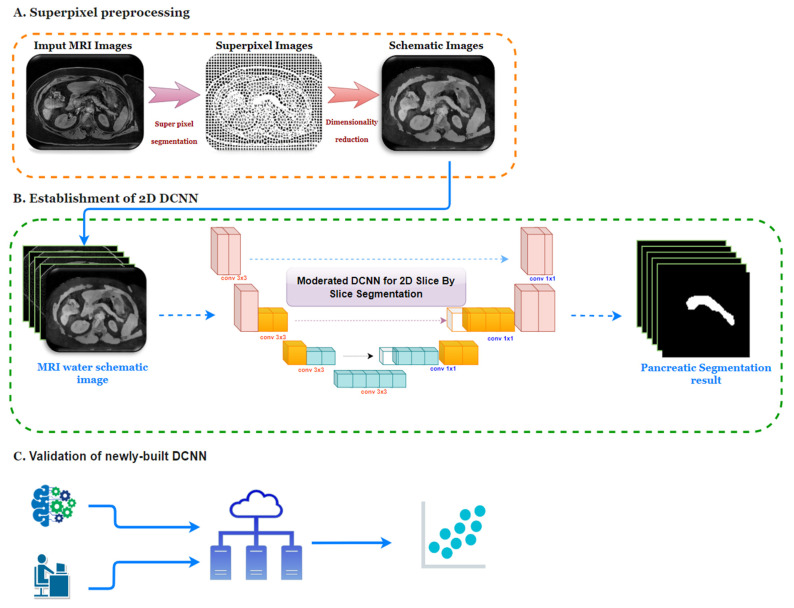
**The overall workflow of the pancreatic segmentation.** (**A**) MR images superpixel preprocessing; (**B**) 2D superpixel DCNN build-up via MR water images; (**C**) validation of the newly built DCNN with MRI obtained from 10 extra participants from the PAT study.

This paper aims to: (1) evaluate the impact of the pancreatic fat fraction to type 2 diabetes in various ethnicities in New Zealand; and (2) describe developing and validating a modified coarse-to-fine 2D framework for auto pancreatic volume and inner pancreatic fat fraction measurement. We then validated the newly built network on 10 extra participants by employing clinical manual calculation and our DCNN. An overview of the segmentation framework is illustrated in Figure 1. 

## 2. Methods

### 2.1. Study Participants and Datasets

This retrospective study involved abdominal MRI from 148 patients with diabetes and 246 healthy normoglycemia controls with multi-ethnic backgrounds (aged ≥18 years, 167 New Zealand European, 106 Māori/ and Pacific Islanders, 67 Asian, and 54 other ethnicities) from Auckland Central Hospital, New Zealand, between August 2015 and October 2019. The 10 extra validation participants were from our PAT study, which details the molecular biomarkers to change before and after bariatric surgery. All participants’ details are summarized in Table 1. All participants underwent abdominal MRI at the Center for Advanced MRI (University of Auckland) on a MAGNETOM Skyra scanner (Siemens, Erlangen, Germany) with a field strength of 3·0 Tesla. Participants were excluded if they had general contraindications for MRI (such as metallic foreign body or electronic device implantation). Exclusions also included pregnancy, malignancy, coeliac disease, cystic fibrosis, chronic pancreatitis, and any history of acute infectious or inflammatory conditions requiring medical evaluation or treatment three months before the study date. All participants included in the study provided written informed consent. Our previous published datasets containing anthropometric measurement and pancreatic fat fractions from MRI readings and specialists were used as a reference for the newly trained DCNN [37]. The independent validation set was derived from additional data from 10 participants who were each scanned twice at the same center. The Health and Disability Committee approved these study protocols. 

### 2.2. Image Preprocessing 

Participants’ water and fat abdominal MR images were included and converted to nifti. format using MRICroGL and pruned to 512×512 pixels, with an average thickness of 3 mm. We selected 2364 MRI water images (1020 of patients and 1344 of control) with recognizable pancreas imaging and manually labeled them using LabelMe [38] for building up DCNN [39]. We used 315 participants’ images for training and 79 data sets for testing (approximately 80:20 ratio). 

MRI modality presents more details in soft tissues due to the slow imaging speed, and the relatively low resolution of MR images often introduces more boundary artifacts for the pancreas. Therefore, preprocessing the images to strengthen the contrast between organ tissue boundaries is essential. Superpixel segmentation is the method that gathers the reduced dimensionality pixels with similar color, brightness, and texture, to generate a visual summary image. It enhances the contrast between the boundaries of different tissues and dramatically increases the accuracy of pancreas recognition. In this study, image preprocessing includes two stages: superpixel segmentation and image dimensionality reduction. 

#### 2.2.1. Superpixel Segmentation

We employed the latest optimized superpixel segmentation method, linear spectral clustering (LSC) for pancreatic image preprocessing. It is regarded as the best-performing superpixel segmentation acting on three-channel natural images [40]. We adjusted the algorithm of the LSC to fit the single-channel MR image task and compared it with simple linear iterative clustering 0 (SLIC0), which was announced as the latest method for superpixel segmentation of medical images [41]. The LSC acting on three-channel natural images was with the following pixel distance calculating formula:

(1) $$D\left(p,q\right)=c_{s}^{2}\left(cos\frac{\pi }{2}\left(x_{p}-x_{q}\right)+cos\frac{\pi }{2}\left(y_{p}-y_{q}\right)\right)+c_{c}^{2}\left(cos\frac{\pi }{2}\left(g_{p}-g_{q}\right)\right)$$

In the formula *D* represents the distance between pixels, *p* and *q* are two different pixels, *c_s_* is the spatial proximity, *c_c_* is the color similarity, *x* and *y* are the spatial positions of pixels, and *g* is the grey scale value of pixels.

Compare this with the pixel distance measurement formula in the original LSC:

(2) $$d_{s}=cos\frac{\pi }{2}\left(x_{p}-x_{q}\right)+cos\frac{\pi }{2}\left(y_{p}-y_{q}\right)$$

We keep the same pixel spatial distance value, d*s*, and change the pixel color distance value from

(3) $$d_{C_{1}}=cos\frac{\pi }{2}\left(l_{p}-l_{q}\right)+2.55^{2}\left(cos\frac{\pi }{2}\left(a_{p}-a_{q}\right)+cos\frac{\pi }{2}\left(b_{p}-b_{q}\right)\right)$$

to

(4) $$d_{C_{2}}=cos\frac{\pi }{2}\left(g_{p}-g_{q}\right)$$

To get better pancreas segmentation performance on restricted GPU, the variables *l, a*, and *b* in these formulae represent the color scale values of pixels, which were replaced by grayscale values. By adjusting the ratio, *r*, of *c_s_* and *c_c_*, the improved LSC method can generate superpixels with higher compactness (*CP*) and boundary recall (*BR*).

(5) $$r=c_{s}^{}/c_{c}^{}$$

The larger the *r*-value, the closer the spatial pixels cluster together, which generates superpixels with high *CP* but low *BR*. Conversely, low *r* values render pixels with low CP but high BR. To get the superpixels with both high CP and BR, we propose a new index as follows:

(6) I=1−BR/CP

The CP and BR would be balanced when *I* reach a minimum positive value. We used this study’s simulated annealing (SA) method to determine the r-value.

We used the evaluation index of superpixel segmentation to compare the performance of our improved LSC with SLIC0. Index elements used are under segmentation error (*UE*), boundary recall (BR), and achievable segmentation accuracy (ASA) [42]. These individual elements can be derived from the following formulas.

The formula for *UE*:

(7) $$UE=\frac{Us}{Rs+Os}$$

In this formula *O_S_* represents the observed number of segmented pixels that should not have been presented; *R_S_* is the theoretical number of pixels; *U_S_* is the number of pixels that did not appear in the actual segmented image that, in theory, should have been presented.

The formula for BR:

(8) $$BR\left(S,G\right)=\frac{TP\left(S,G\right)}{TP\left(S,G\right)+FN\left(S,G\right)}$$

*G* and *S* represent the reference and actual boundaries of superpixel segmented images when given a ground truth, respectively. According to Equations (2) and (4), the value of maximum pixel distance is 1. *TP* is the true value of the pixel number of S that overlaps G within the range of d. FN is the opposite value of TP. 

The formula for *ASA*:

(9) $$ASA=\left(1-\frac{|Rs-Ts|}{Rs}\right)\times 100\%$$

*Rs* represents the manually segmented reference area the expert marks; *Ts* is the area from the trained DCNN. 

#### 2.2.2. Dimensionality Reduction of Images

We adjusted the images by reducing the dimensionality and generated their corresponding visual summary maps that only showed the features between superpixels. Combined with the idea of average pooling in deep learning, we input the DCNN, a calculation of averaging gray values of all pixels in each superpixel, and then reassigned values to each gray value to generate a schematic diagram. The preprocessed abdominal schematic diagram enhanced the display of the separability between organs and tissues, which is conducive to the segmentation of the pancreas. 

### 2.3. DCNN Establishment and Performance Evaluation

**Figure 2:**
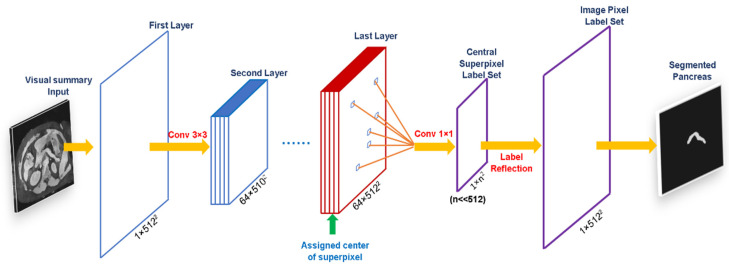
The framework of the DCNN. The assigned center of superpixels accelerated the speed of DCNN.

We fine-tuned an existing U-net model and trained it to a new single-branch DCNN architecture from scratch to converge based on a large amount of artificially labeled training data of MRI water images. To increase the DCNN accuracy, we transferred learned kernels in the bottom layers of the VGG-16 network to our domain, which has been highly discriminative with a stable training convergence [43]. U-net models have produced extraordinary results in medical image segmentation combining the information from low and high medical image layers, which help improve accuracy and extract complex features, respectively. In the last layer of DCNN, it performs 1×1 convolutional classification, which slows its inference procedure significantly. To address this problem, we proposed an acceleration method shown in Figure 2. The DCNN only performs convolutional classification on the labeled superpixel at the center position, representing the same pixel cluster with identical characteristics. This method ensured both the network speed and its classification performance. 

The training was conducted on a workstation with Inter i9-11900k CPU 2080 NVIDIA Ti GPU using Python 3.6.0 and deep learning packages TensorFlow and Keras. The DCNN training process included continuous iterations of forward followed by backward propagation. The forward propagation involves feeding input images and returning corresponding output sets of per-pixel predictions within the pancreas. The segmentation accuracy of each sample was evaluated by the Dice Similarity Coefficient (*DSC*), which measures the overlap between the user-annotated pancreas and the DCNN-predicted masks (shown as *X* and *Y*, respectively):

(10) $$DSC=\frac{2\times |X\cap Y|}{|X|+|Y|}$$

To further validate the novel DCNN, we applied it to 10 additional newly-recruited participants with prediabetes from our latest clinical research (Table 1). Two independent, experienced researchers segmented pancreatic volume and fat fraction, and their result was then compared with the machine-derived results. The time consumption and how closely the segmentation results are related were observed.

### 2.4. Principles of Measuring Pancreas Volume and Pancreatic Fat Deposition

#### 2.4.1. Phantom Study 

**Figure 3:**
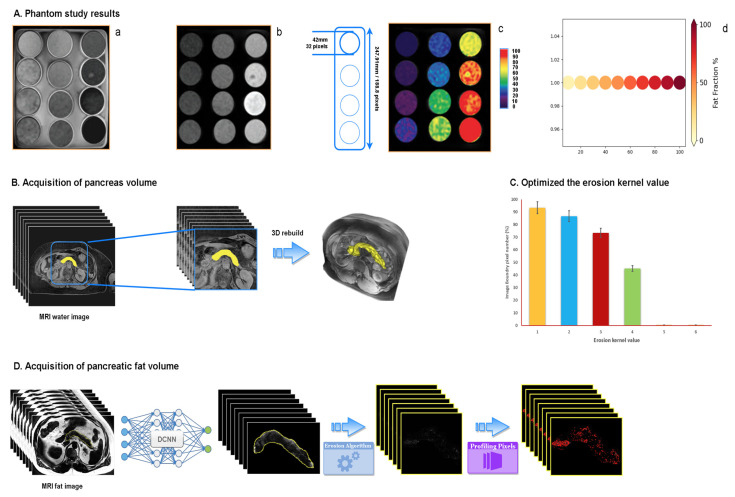
Processing of pancreatic tissue scan and its inner fat volume acquisition. (**A**) Phantom results under the same MRI conditions: (**a**) water phase MRI scan; (**b**) fat phase MRI scan; (**c**) heatmap of fat gradient generated by app in MRS; (**d**) digital converted fat fraction gradient generated by python pillow algorithm for machine learning; (**B**) method of acquisition of pancreas volume and its 3D rebuilt result; (**C**) optimization of erosion kernel value; and (**D**) process of acquisition of pancreatic fat volume from MRI fat image to profiling pixels.

We conducted a phantom emulsion series of readings to convert the MRI pixel information into actual dimensions and calibrate the proportion of fat tissue for the machine. We used vegetable (soy) oil and distilled and undoped water to make a homogeneous emulsions series to evaluate the accuracy of each quantification result. This method was fully described by Bernard [44,45]. We added 0–100% fat volume fractions and lecithin (1% by weight from Sigma) in 100 mL bottles. To stabilize the emulsions, we applied agar gel (3% by weight) and dioctyl sulfosuccinate sodium salt to the system. The emulsions were prepared slowly over a heat-stir plate and subsequently cooled down to room temperature allowing the suspension to stay evenly distributed. The bottles were then placed in a container with solid agar and processed in the MRI machine under the same condition that operated on participants. Single voxel MRS was performed on ten emulsions with a fat fraction of 10–100% fat with the same scan parameters as repetition time = 4 s, echo time = 23 ms, and bandwidth = 2.5 kHz. The phantom results are presented in Figure 3A.

#### 2.4.2. Acquisition of Pancreas Volume

We input the water phase MR images into the well-trained DCNN and enabled our newly-trained framework to calculate the segmented area for further accumulating the total volume. The segmental volume was calculated as the product of the area of each pancreatic slice (generally, there were 6–9 pancreatic MRI images for each participant) and the thickness (3 mm), as indicated by the Cavalieri principle: [45] The pancreatic 2D images were stacked and restructured into a 3D model (Figure 3B).

(11) Volume in vivo=∑Volumes of all pancreatic slices

#### 2.4.3. Acquisition of Pancreatic Fat Volume

Manual operation quantifies the area of pancreatic fat pixels within the entire pancreas on fat images. The values were noted by Image J (National Institutes of Health, Bethesda, MD, USA) with automatically evaluated pixel percentages. To reduce inter-observer and intra-observer variability, we manually removed the pixels with fat percentage values of <1% and >20%, representing histologically verified blood vessels, ducts, or visceral fat [24]. 

We enabled our DCNN to have the identical function as manual measurement. Our newly-built DCNN can pick up MRI fat images with a redundant boundary. To avoid contamination of the redundant border and improve the calculation performance, we employed the erosion algorithm (X Θ B = X − b = {z: (B + z) ⊆ X}) in OpenCV to remove the extra boundary pixels from the segmented images. The erosion algorithm computes a local minimum over a given kernel value, which we adjusted in our DCNN until we achieved a satisfactory segmenting outcome. The stability of the highest performance was established when the kernel value was adjusted to 5 (Figure 3C). The phantom study aligned the brightness of each pixel on MRI to its corresponding actual fat percentage. The performance of the erosion algorithm and machine pixel labeling on MR fat images is shown in Figure 3D. 

### 2.5. Statistics

The SciPy algorithm in Python was employed to do all statistical analyses. An independent Student *t*-test was used to reflect the difference in detecting pancreatic fat fraction in diabetic patients and healthy normoglycemia controls and the DSC scores under various training conditions. 95% Cl of the critical *p*-value was considered significant when it was less than 0.05. Linear regression was performed to validate the segmentation performance of specialists and newly-built DCNN.

## 3. Results

### 3.1. Evaluation of the Correlation between the Pancreatic Fat Fraction and Type 2 Diabetes in Various Ethnic Groups

**Figure 4:**
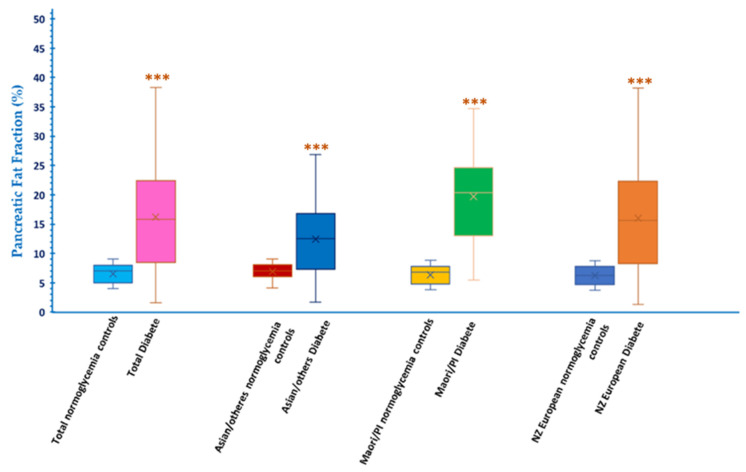
The comparison of pancreatic fat fraction between normoglycemia control and T2D in multiple ethnic groups. Each group is significantly different with *p* < 0.01 ***.

The manually measured pancreatic fat fraction data was organized into multiple groups based on ethnicity and T2D condition. Student *t*-test was employed to analyze the difference between groups. The results are shown in Figure 4. The pancreatic fat fraction is significantly different between total normoglycemia controls and diabetes with a t value of −13.0799, *p* < 0.01. The t values of the difference between normoglycemia and diabetes in various ethnic groups are −8.2767 for NZ Europeans, −9.9537 for Māori and Pacific Islanders, and −4.9576 for Asian and others. All groups are with a value of *p* < 0.01. Notably, the pancreatic fat fraction within normoglycemia controls was similar. However, the pancreatic fat fraction among patients with diabetes was significantly different.

### 3.2. The Performance of Superpixel Segmentation

#### 3.2.1. Results of the Comparison of Improved LSC and SLIC0

**Figure 5:**
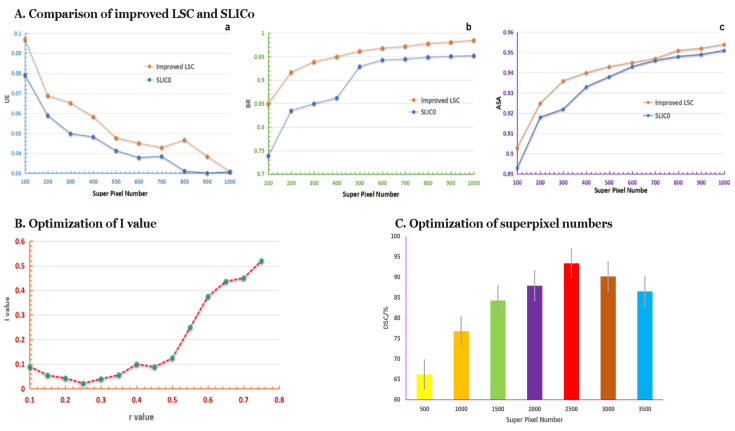
Variation of different numbers used in improved LSC and the segmentation results of MRI imaging. (**A**) Comparison between the improved LSC and SLIC0 with superpixel segmentation indexes; a, b, and c are the comparison of UE, BR and ASA, respectively. (**B**) The minimum I value was set at r = 0.25. (**C**) Evaluation of the best superpixel number for segmentation. The highest DSC was acquired when the superpixel number reached 2500.

We adjusted the LSC with the new method of distance measurement. The performance comparison of improved LSC and SLIC0 is shown in Figure 5A, through which we can see the enhanced LSC performed better than SLIC0 in UE, BR, and ASA indexes. The results showed that the LSC method better satisfied the actual requirements of superpixel segmentation of medical images. 

#### 3.2.2. Request r-Value by Improved LSC Method

The segmented result of superpixel on medical images should be expected to have both high BR and CP values. We adjusted the ratio of c_s_ and c_c_ values to achieve this target by the improved LSC method. According to the result of simulated annealing, we selected the r-value range at [0.05, 1] and calculated the *I* value accordingly. The correlations of different r values of superpixel segmentation and *I* values are shown in Figure 5B, from which we can see the optimum and minimum *I* value was derived when r = 0.25, which achieved the balance between CP and BR values.

#### 3.2.3. The Optimum Number of Superpixels

We preprocessed the medical images and got their corresponding superpixel schematic shots to get the best pancreas segmentation result. The processed images were then input into the DCNN for training and testing until the model optimized the DSC value. The number of obtained superpixels was a crucial factor that defined the quality of segmentation. Training DCNN (for 20 epochs) resulted in adjustment to the number of superpixels on preprocessing images at values of 500, 1000, 1500, 2000, 2500, 3000, and 3500, respectively. The results suggest that DSC tends to be optimized when the superpixel number is 2500, as illustrated in Figure 5C.

### 3.3. Evaluation of the Preprocessing at Different Epochs

**Figure 6:**
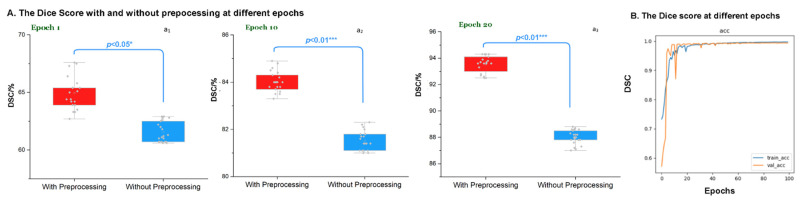
**DSC comparison of the modified DCNN at different epochs with and without preprocessing of the superpixel.** (**A**) The DSC comparison at different epochs, *p* values showed that they were significantly different at different epochs: a_1_, a_2_, and a_3_ are the results at 1, 10, and 20 epochs with *p* values of 0.05, 0.01, and 0.01, respectively. (**B**) The Dice Score for accuracy at different epochs, after 20 epochs, the framework became stable.

We compared the average Dice Score values of our DCNN with and without preprocessing images at various epochs; the results showed that higher average Dice Score values came out from the DCNN with the input of the preprocessed images (Figure 6A). The accuracy curve based on Dice Score showed that the weighted network fit in preprocessed images became stable after 20 epochs (Figure 6B). Framework performance also stabilized after 20 epochs by feeding no preprocessing images. Considering saving time, we set the optimum conditions for our DCNN as training for 20 epochs with the number of superpixels at 2500.

### 3.4. Segmentation Integrity of Novel DCNN 

**Figure 7:**
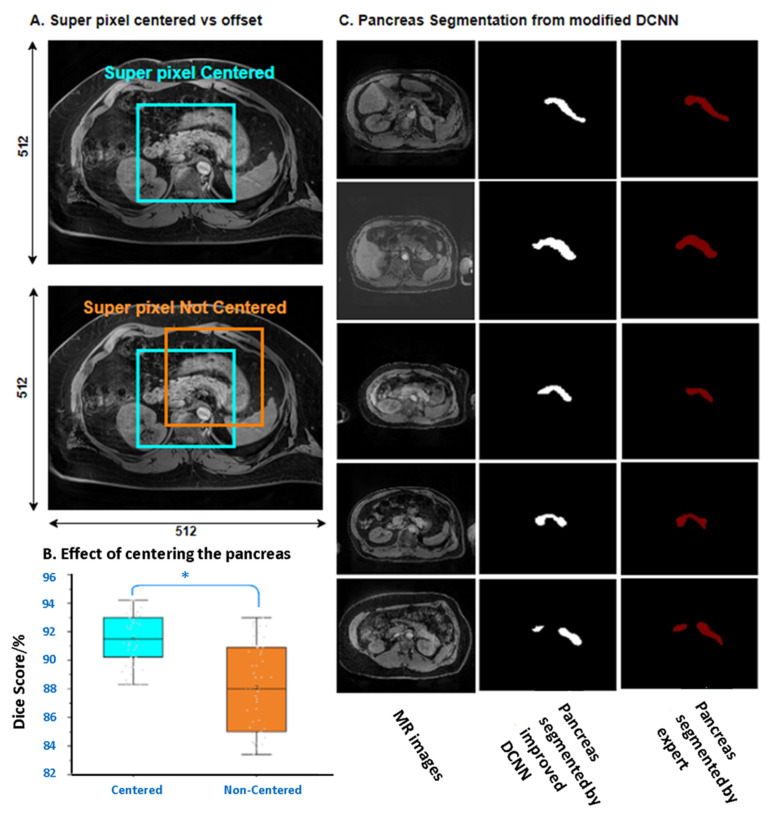
(**A**) Effect of superpixel centering of modified DCNN. (**B**) Performance summary comparing the superpixel segmentation methods of pancreas images, centered and non-centered. (**C**) The pancreatic segmentation results of DCNN and expert manual operations. *p* < 0.05 *.

The performance comparisons between superpixel centered and not centered are shown in Figure 7A,B. The results showed that centered superpixel on images could significantly increase the DSC of the DCNN. The corresponding DSC for 20 epochs reached up to 91·2%. Overall, the DSC value from the novel DCNN met the actual requirements, and the integrity of the pancreatic organ from testing images is shown in Figure 7C. The DSC values of recent frameworks are summarized in Table 2 for a general comparison. 

### 3.5. Independent Validation of the Novel DCNN

**Figure 8:**
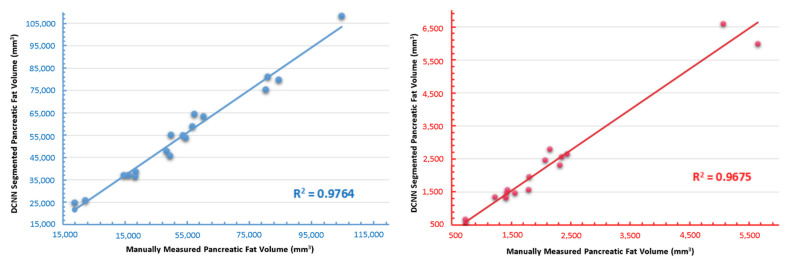
Validation of the integrity of newly trained DCNN. Performance validation measuring pancreas and pancreatic fat volume between expert manual operation and DCNN. The R^2^ values were 97.64% and 96.75%, respectively.

The newly-built DCNN performance was validated with specialist manual segmentation on newly recruited patients. The regression graph reflected the correlation between the two segmenting methods regarding pancreas volume and pancreatic fat fraction (Figure 8). The R^2^ values of pancreas volume and fat fraction were 0.9764 and 0.9675, respectively. We can roughly conclude that the segmentation results derived by the newly-generated DCNN were reliable compared with the manual operation. Further, the DCNN shortened the time consumption dramatically. The average time for measuring each patient was from 1.5 h via manual process to 5 s using the DCNN.

## 4. Discussion

Our study provides positive evidence for the clinical concept that inner-pancreatic fat correlates with T2D. The result solidified the idea of developing a DCNN model for an auto-measuring pancreatic fat fraction for type 2 diabetes diagnosis. We compared consuming time on manual and auto segmentation methods. It achieved an accuracy approach comparable to manual grading, obtained at a much greater speed (5 s for DCNN analysis vs. 1.5 h for manual grading). This study also provides proof of the DCNN-based analysis of pancreatic fat and pancreatic volume among participants of different ages, BMIs, and ethnicities. Because the training, validating, and testing sets shared no common participants, the excellent performance of the DCNN could not be attributed to overfitting. Ethnicity and BMI are key factors that influence the imaging characteristics of the pancreas, including pancreatic fat [49,50,51]. To ascertain the potential generalizability of the DCNN in recognizing pancreatic fat and volume in people of different ethnicities and body sizes, we tested the DCNN trained on a multi-ethnic group of participants with an external dataset of participants. These participants differed substantially from the initial training set in having a higher BMI. MR images were taken before and three days after bariatric surgery when type 2 diabetes status was present and absent, respectively, correlating to a significant reduction in pancreatic fat and volume over this short peri-operative time interval. The excellent performance of the DCNN on the external dataset suggests a good representation of BMI and ethnicity in the training set images. 

Recent studies showed that pancreatic fat deposition and volume are causally linked to developing type 2 diabetes and other metabolic diseases [2,13]. Thus, accurate quantification of pancreatic parameters is vital for clinical use. The pancreas volume and fat fraction measuring protocols are software-based manual segmentation developed from 3D IDEAL MRI scans [24]. However, due to the anatomical features of the pancreas, which is comparatively small, it is challenging to localize the measuring center on MR images [52,53]. The post-MRI manual expert-based methods currently define pancreatic quantification. This method provides a more accurate output than the direct reading from MRS. [24]. However, it requires an experienced specialist to carry out extensive freehand drawing around ROI pixels, which is incredibly time-consuming; hence we developed this DCNN to automate pixel definitions for broader use. Some framework performances are listed in Table 2 for a rough comparison. However, it is of minimal use to make the comparison between frameworks based on different algorithmic strategies. Due to a mere handful of DCNNs for pancreas segmentation being on the market, we have presented them for reference purposes only.

Prior studies have rarely characterized the pancreas for either fat deposition or volume due to high variability in size and location among patients [54,55]. We introduced the superpixel segmentation method to our DCNN and preprocessed the medical images. We innovated the calculation index of pixels to improve the LSC algorithm, which turned superpixel segmentation acting on three-channel natural images to only one-gray-channel MRI images and adapted it to the evaluation of medical images. Results showed that the superpixel clarified the boundary of the pancreas, making recognition easier for the DCNN. The comparison results of user-based and automatic segmentation showed that our DCNN was particularly well-suited to this task since it computes pancreas presence and location on a superpixel basis with a multi-resolution approach possessing a U-net-base architecture. Compared with the user-performed segmentation, this method produced results with lower error ranges and saved considerable time on calculations. With this method, it would be more feasible to measure pancreatic fat deposition and size for extensive data sets and accelerate metabolic disease research. With limited further training, it theoretically can also be compatible with other types of medical images like CT and ultrasound.

There are still some limitations to the current work: (1) this study included images obtained from a standardized MRI protocol and scanner, but whether the performance of the DCNN generalizes to data obtained using various scanners and protocols should be further investigated; (2) the algorithm may only be valid on fat infiltration pancreas. However, the DCNN is loyal to the clinical segmentation method, and fat infiltration on the pancreas is also an essential index of various metabolic diseases diagnosis; (3) in the validation process, we only recruited 10 new participants due to the impact of COVID-19. It may raise the risk of overfitting based on such a small dataset. Further validation needs to be conducted by including more participants; (4) though the novel DCNN was built upon the two-dimensional (2D) dataset, it could be possible to be extended to 3D prediction, which helps further with pancreatic volume evaluation. However, it is challenging to expose the fat pixels inside the pancreas. An appropriate algorithm to overcome the boundary clearance and inner pancreatic fat pixel accumulation needs to be deliberated; and (5) a more extensive training and validating set could provide the DCNN with further stability and efficiency in the proposed approach. 

## 5. Conclusions

We successfully developed one artificial intelligence model to measure pancreas volume and fat deposition from MR images. We first copied the manual measurement method of the pancreas into the DCNN. We also initially involved a preprocessing method of a superpixel algorithm on MR images to assist with pancreatic boundary recognition. The overall mean DSC is 91.2%, the highest known DCNN of the same type. Our future work will focus on the 3D model establishment and obtaining more training datasets to improve accuracy. It will assist with large-scale automated pancreas volume and fat deposition processes in research and clinical applications. It also has the potential to be set up in a cloud to provide free service to encourage more pancreas imaging research. 

## Figures and Tables

**Table 1:** Detail of the participants.

Characteristics	DCNN Training and Testing				Independent Validation			
	Total	NZ. European	Māori/Pl	Asian/Others	Total	NZ. European	Māori/Pl	Asian/Others
No. of participants	394	167	106	121	10	4	4	2
Age (years)	47.8 ± 1.2	52 ± 2.4	55.3 ± 1.9	35.7 ± 2.3	38.8 ± 0.8	37.7 ± 1.1	32.9 ± 0.6	39 ± 1.2
Sex								
Male	249	98	71	81	4	2	1	1
Female	145	69	35	40	6	2	3	1
BMI.	28.8 ± 0.7	28.1 ± 0.5	33.7 ± 1.3	25.4 ± 1.0	35.6 ± 1.6	35.5 ± 1.2	38.8 ± 1.8	29.3 ± 0.6
Pancreatic fat fraction% (MRS)	31.2 ± 2.0	29.7 ± 1.5	38.4 ± 2.8	26.8 ± 2.2	46.2 ± 1.9	39.3 ± 1.4	46.8 ± 1.7	52.5 ± 2.6
Pancreatic fat fraction% (Manual)	8.4 ± 0.6	7.6 ± 0.4	9.9 ± 0.8	8.4 ± 0.6	8.5 ± 0.5	8.8 ± 0.3	8.6 ± 0.6	7.6 ± 0.5

**Table 2:** Mean DSC values of recent frameworks for pancreatic segmentation.

Method	Mean Dice Score/%
Attention U-net [46]	81.5
3D FCN [28]	76.8
RSTN [35]	84.5
Graph-based decision fusion [31]	76.1
Lightweight DCNN modules [47]	85.6
Spatial aggregation [29]	81.3
Fixed-Point [40]	83.2
Bayesian Model [48]	85.3
Our DCNN	91.2

## Data Availability

The datasets used and/or analyzed during the current study are available from the corresponding author on reasonable request.
